# Insights into parental perspectives: Children's eye care in Saudi Arabia

**DOI:** 10.1016/j.heliyon.2024.e41179

**Published:** 2024-12-12

**Authors:** Sokinah N. Al Musalami, Reem J. Al Qasim, Bayan S. Alshuhayb, Abdulaziz I. Al-Somali

**Affiliations:** a Eastern Health Cluster, Dammam, Saudi Arabia; b Prince Sultan Military Medical City, Department of Ophthalmology, Riyadh, Saudi Arabia; c King Saud University, Department of Ophthalmology, Riyadh, Saudi Arabia; d King Faisal University, Department of Surgery, Ophthalmology Unit, Saudi Arabia

## Abstract

**Purpose:**

Despite the concerning growth in the number of children with preventable or treatable causes of blindness, parents and/or children often do not notice many eye problems due to the lack of adequate knowledge about them. Considering the lack of updated relevant literature on this topic, this study aimed to gain insights into parental perspectives regarding children's eye health and the barriers that prevent them from promptly addressing these issues.

**Methods:**

A cross-sectional online survey was randomly distributed to Saudi parents of children aged 0–18 years from February 2022 to April 2022.

**Results:**

A total of 1265 parents (mean age = 36.10 ± 10.043 years) completed the questionnaire, and 61.3 % obtained high knowledge scores. The factors associated with high knowledge scores included an educational level above high school, employment in the healthcare industry, and having a child with an eye disease.

Two-thirds of the parents did not take their children for periodic eye examinations. However, parents from the central region, healthcare workers, and those having a child with an eye disease were more likely to have their children's eyes checked regularly. Moreover, 40 % of parents tended to believe that routine eye checkups were unnecessary if their children had seemingly normal eyes and no eye complaints.

Concerning corrective spectacle use, parents from the northern region and those with an educational level of high school or less were more likely to reject it. Our findings also showed that 25 % of parents believed that eyeglasses may limit their children's daily activities.

**Conclusion:**

Parental knowledge of children's eye care in Saudi Arabia was acceptable.

## 1 Introduction

The early years of life are of utmost importance for vision development, both anatomically and functionally [1]. Any disturbances in eye focus or alignment during this critical period can lead to visual impairment or blindness. According to a study conducted by the World Health Organization (WHO) in 2010, 1.421 million and 17.518 million children aged 0–14 years were blind and had low vision, respectively [2]. Fortunately, with early detection and timely intervention, most eye conditions that cause visual impairments in children can be prevented or treated [3]. According to the WHO, 40 % of childhood blindness is preventable and treatable [4]. However, because of a lack of knowledge about common eye problems and their possible presentations in children, these problems can go unnoticed by parents and caregivers [3,5].

Prevention, early detection, and prompt treatment of vision problems must be undertaken not only because of the high prevalence of these problems but also because of their major negative impact on a child's quality of life [3]. Children with poor vision can experience lifelong consequences, including difficulties in performing everyday activities, engaging in social life, attaining an adequate level of education, and finding employment opportunities. Being a parent of a visually impaired or blind child is not an easy job either. Such children require constant social, emotional, physical, and financial support to help them adapt to their visual impairments. This explains why parents and other caregivers of children with eye diseases are more prone to mental and physical exhaustion [3]. However, these consequences are avoidable through proper education of caregivers about common childhood eye conditions, the importance of periodic eye examinations, and appropriate eye care [3].

The prevalence of amblyopia in Saudi Arabia (KSA) has been reported to be 0.5 % in Riyadh, 3.9 % in Qassim Province, 1.3 % in Jeddah, and 9.1 % in Dammam, and the pooled prevalence in KSA is 2.3 % [[6], [7], [8], [9], [10]]. In a recent systematic review, the most frequent form of amblyopia was identified as refractive amblyopia (65.1 %), followed by strabismus amblyopia (29.4 %) [10]. Moreover, in a systematic review conducted in KSA, the pooled prevalence of uncorrected refractive errors among children was 16.8 % [11]. These findings raise concerns about parental knowledge and practices regarding children's eye healthcare and highlight the need to investigate the barriers preventing parents from seeking appropriate and timely eye care for their children. However, only a few studies have been conducted on this topic in Saudi Arabia. Most of these studies have concluded that the level of parental knowledge of children's eye care is unsatisfactory and highlighted the need for educational programs [3,5,12]. However, further studies in this field are warranted, since one of these studies had an inadequate sample size (97 participants) while the others were conducted in a single city. Since Saudi Arabia is a large country with many regions and cities, a more extensive study with a larger sample size is required to obtain more accurate results. Therefore, this study aimed to determine parents' knowledge, attitudes, and practices regarding children's eye healthcare across all regions of Saudi Arabia and to explore the major barriers that prevent parents from seeking regular eye checkups for their children.

## 2 Methods

### 2.1 Study type

This community-based, retrospective, cross-sectional study was conducted in Saudi Arabia from February 2022 to April 2022.

### 2.2 Population and sample size

The target population consisted of Saudi parents with children aged 0–18 years. The inclusion criteria were Saudi parents (not necessarily couples) with children aged 0–18 years. Non-Saudi parents and parents with no children aged 0–18 years were excluded. Using a 95 % confidence level and 5 % margin of error, the minimum sample size was calculated as 385 using Raosoft. In total, 1265 parents completed the questionnaire.

### 2.3 Measurement tools

The questions were adapted from a questionnaire used in a study by Donaldson, and the other questions were formulated on the basis of the authors’ experiences [13]. Minor modifications were made to ensure that the survey was suitable for the study sample.

The final questionnaire evaluated five aspects.

1. Demographic Data and Living Circumstances: This aspect was evaluated by collecting basic demographic information and data regarding living conditions.
2. Parents' Knowledge of Common Children's Eye Diseases: Parents' knowledge was evaluated using 18 questions covering strabismus, refractive errors, amblyopia, and low vision. Each correct answer was scored one point, and a score above nine indicated sufficient knowledge.
3. Parents' Attitudes Regarding Children's Eye Diseases: Parents' attitudes were evaluated using direct questions assessing the usage of corrective spectacles and governmental healthcare services.
4. Parents' Responses to Children's Eye Complaints: This aspect was evaluated using scenario-based questions where parents indicated their likely responses.
5. Parents' Practices and Sources of Knowledge Regarding Children's Eye Diseases: This factor was investigated through questions about the frequency of eye checkups and sources of information on eye health.

All questions were translated into Arabic, the primary language of the study population. Before distribution, the questionnaire was tested by five ophthalmologists to ensure content validity, and a pilot study was conducted with 10 % of the calculated sample size (approximately 39 parents) in a local community center. Based on the feedback from the pilot study, minor modifications were made to clarify some questions and improve the flow of the questionnaire. The final version of the questionnaire was then distributed electronically.

### 2.4 Institutional Review Board approval and data collection

This study was approved by the Biomedical Research and Ethics Committee of King Faisal University. After receiving approval and validation from the Institutional Review Board, the questionnaire was electronically distributed to the community using Google Forms links via different social media platforms. Fourteen data collectors assisted in the data collection. The estimated time required to complete the form was at least 5–6 min. After obtaining informed consent, the participants were included in the study. All data were kept confidential, and no participants were identified by name. The questionnaire was distributed to 1399 Saudi parents, and after excluding 134 responses in accordance with the inclusion and exclusion criteria, the remaining 1265 responses were included in the analysis.

### 2.5 Statistical analyses

Data analysis was performed using IBM Statistical Package for the Social Sciences software version 22. The data are not normally distributed. Normality was tested using the Kolmogorov–Smirnov test. Categorical variables were expressed as frequencies and percentages. The chi-square test was used to verify the associations between categorical variables. The Mann–Whitney *U* test was used to assess the relationships between continuous and categorical variables. The level of significance was set at *p* < 0.05.

## 3 Results

### 3.1 Demographic characteristics

A total of 1265 parents (mean age = 36.10 ± 10.043 years) completed the questionnaire. Among these, approximately 68 % were mothers, and more than 80 % had studied above high school. Only approximately one-fifth of the participants were working in the healthcare industry. Almost half of the participants had 1-2 children, while 40 % of the respondents had a child who had been diagnosed with an eye disease. The demographic characteristics of the respondents are summarized in Table 1.

**Table 1:** Demographic Characteristics of the participants and the awareness score (N = 1265).

Variable	Category	Data (%)
***Age***	*Maximum*	67 years old
	*Median*	35 years old
	*Minimum*	17 years old
	*Mean (±Std Dev)*	36.10 (±10.043) years old
***Gender***	*Male*	404 (31.9 %)
	*Female*	861 (68.1 %)
***Educational Level***	*Advanced degree*	154 (12.2 %)
	*Bachelor*	740 (58.5 %)
	*Diploma*	147 (11.6 %)
	*High school*	169 (13.4 %)
	*Less than high school education*	55 (4.3 %)
***Saudi Arabian Region***	*Central Province*	243 (19.2 %)
	*Eastern Province*	366 (28.9 %)
	*Northern Province*	169 (13.4 %)
	*Southern Province*	227 (17.9 %)
	*Western Province*	260 (20.6 %)
***Number of children***	*1–2*	657 (51.9 %)
	*3–4*	379 (30 %)
	*5–6*	118 (9.3 %)
	*More than 6*	111 (8.8 %)
***Type of work***	*Healthcare industry*	260 (20.6 %)
	*Other than healthcare industry*	1005 (79.4 %)
***Has one of your children been diagnosed with children eye disease?***	*Yes*	518 (40.9 %)
	*No*	747 (59.1 %)
***Awareness Score***	*Low awareness score*	490 (38.7 %)
	*High awareness score*	775 (61.3 %)

### 3.2 Parents' knowledge of children's eye diseases

In the assessment of parental knowledge, nearly 61 % of the parents showed a high knowledge score. An educational level above high school (*p* = 0.004), working in the healthcare industry (*p* < 0.0001), and having a child with an eye disease (*p* = 0.001) were associated with higher knowledge scores among parents. However, the parents’ knowledge scores showed no significant associations with parent age, sex, number of children, or region.

### 3.3 Parents' attitudes regarding children's eye diseases

**Fig. 1:**
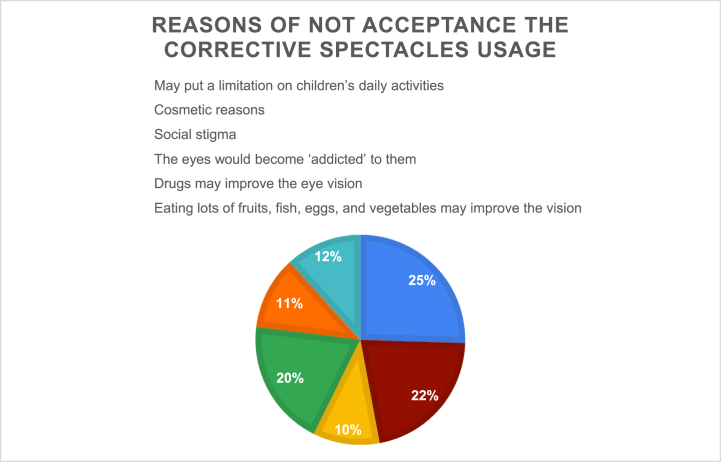
Reasons for not acceptance the corrective spectacles usage.

In the assessment of parental attitudes, most participating parents considered the use of corrective spectacles for their children's eye health to be acceptable (92.4 %). However, parents from the northern region and those with an educational level of high school or lower were more likely to reject the use of corrective spectacles (*p* = 0.004 and < 0.0001*,* respectively). When asked about the reasons for rejection, 25 % of these parents believed that eyeglasses may limit their children's daily activities, while 22 % had only cosmetic concerns [Fig. 1]. When asked about their willingness to attend educational sessions, parents were willing to attend such sessions whenever they became available. Only 48 % of the participants had tried one of the available governmental healthcare services for children's eye diseases. Most of the participants who had tried these services were satisfied with them [Table 2].

**Table 2:** The opinions of the participants about different variables (N = 1265).

Variables	Opinion		
*What is your opinion regarding the usage of corrective spectacles?*	**Acceptable** 1169 (92.4 %)		**Not acceptable** 96 (7.6 %)
*If you tried one of the available governmental health care services for children eye diseases, are you satisfied with it?*	**Yes** 420 (33.2 %)	**Did not try before** 657 (51.9 %)	**No** 188 (14.9 %)
*If there are educational sessions about children eye diseases, would you be willing to attend?*	**Yes** 1053 (83.2 %)		**No** 212 (16.8 %)
*Do you take you children for eye examination periodically?*	**Yes** 435 (34.4 %)		**No** 830 (65.6 %)
*Did you attend previously to any children eye diseases educational sessions?*	**Yes** 238 (18.8 %)		**No** 1027 (81.2 %)
*Did your child's eyes have been examined when she/he was*6 months *old?*	**Yes** 421 (33.3 %)	**Can not recall** 369 (29.2 %)	**No** 475 (37.5 %)
*Do you know how to access an eye test appropriate for your child's age if you had any concerns?*	**Yes** 802 (63.4 %)	**Not sure** 268 (21.2 %)	**No** 195 (15.4 %)

**Fig. 2:**
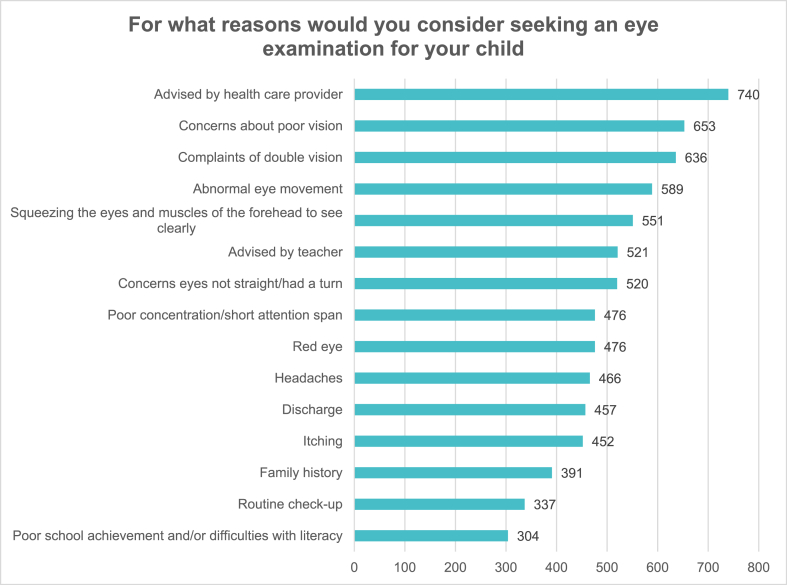
For what reasons would you consider seeking an eye examination for your child.

**Fig. 3:**
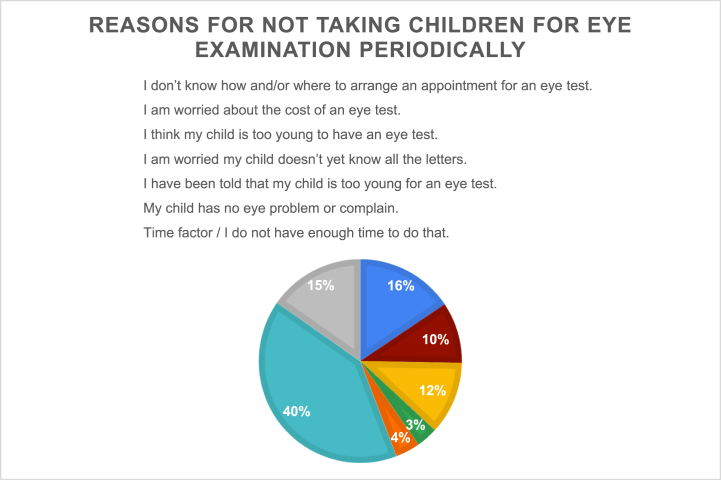
Reasons for not taking children for eye examination periodically.

The reasons for which parents considered taking their children for eye examinations are listed in Fig. 2. The most frequent reasons were advise from a healthcare provider and concerns about poor vision. However, 40 % of parents believed that routine eye checkups were unnecessary if their children had seemingly normal eyes and no eye complaints ]Fig. 3[. Approximately 12 % of the participating parents believed that their children were too young to have their eyes tested. Moreover, while 16 % of the respondents did not know how and/or where to arrange an appointment for an eye examination for their children, while 15 % knew but did not have the time to do so.

### 3.4 Parents' practices and sources of knowledge regarding children's eye diseases

**Fig. 4:**
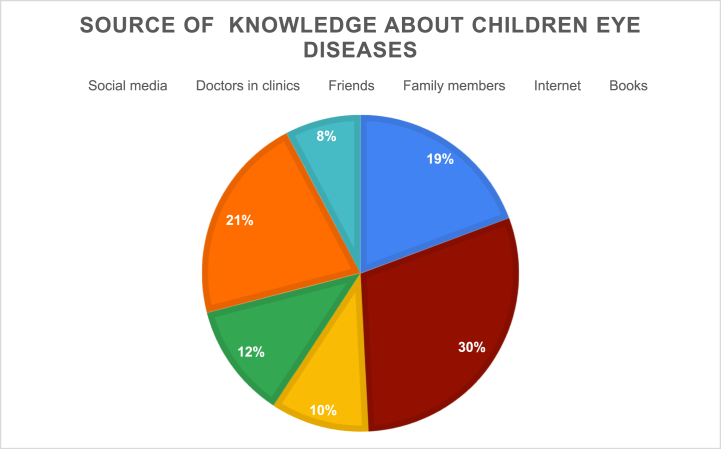
Source of knoweledge about children eye diseases.

Approximately two-thirds of the parents did not take their children for periodic eye examinations [Table 2]. Parents from the central region (*p* = 0.002), those working in the healthcare industry (*p* = 0.013), and those who had children with eye diseases (*p* < 0.0001) were more likely to have their children's eyes checked regularly [Table 3]. More than 80 % of the parents participating in this study had not attended educational sessions on children's eye diseases. Moreover, while 30 % of the parents only obtained information about children's eye diseases from doctors in clinics, 21 % and 19 % relied on the Internet and social media, respectively, as their source of information (Fig. 4).

**Table 3:** Association between eye examination periodically for children and different variables (N = 1265).

Variable	Eye examination periodically for children		X^2^ value	P.value
	No	Yes		
**Gender:**				

*Female*	563	298	0.060	0.849
*Male*	267	137		
**Educational level:**				

*High school education and less*	143	81	0.377	0.536
*Above high school education*	687	354		
**Region:**				

*Central province*	140	103	17.465	0.002
*Eastern province*	255	111		
*North province*	97	72		
*South province*	157	70		
*Western province*	181	79		
**Number of children:**				

*1-2*	417	240	4.151	0.244
*3-4*	264	115		
*5-6*	77	41		
*>6*	72	39		
**Work:**				

*Healthcare industry*	153	107	6.511	0.013
*Other than healthcare industry*	677	328		
**Having a child with eye disease:**				

*No*	575	172	104.121	<0.0001
*Yes*	255	263		
**Age:**				0.827

∗P. value of 0.05 or less was considered significant.

## 4 Discussion

### 4.1 Parents' knowledge regarding children's eye diseases

In the present study, the parental level of knowledge regarding children's eye healthcare was approximately 61 %. Parents with an educational level above high school, a job in the healthcare industry, and/or a child suffering from an eye disease had better knowledge of common childhood eye conditions than parents without these characteristics. Overall, Saudi parents' knowledge of childhood eye care was acceptable. This is especially true when the findings were compared with the outcomes of other local studies in the literature, which were notably lower. Poor knowledge of childhood eye care and diseases was reported by 91.9 % of parents in Riyadh, 78.2 % in Madinah, 72.8 % in Makkah, and 61.1 % in the Aseer region [[12], [13], [14], [15], [16]]. Likewise, Bashaar et al. reported that the overall knowledge score among parents of children attending public schools in Saudi Arabia was 2 out of 7, which was below average [5]. Conversely, another study conducted in Arar City, Northern Saudi Arabia, concluded that 56.7 % of parents had an adequate level of knowledge about pediatric eye diseases [17]. In the present study, the majority (over 80 %) of the parents had an educational level higher than high school, which may have contributed to their better understanding of childhood eye care.

The present study showed no significant association between parents' knowledge level and their age, sex, region, and number of children. However, parents with higher educational backgrounds had better knowledge of eye diseases and eye care for children (*p* = 0.004). This is consistent with the findings of a previous paper published on Saudi parents' awareness of pediatric eye diseases in Riyadh, which concluded that the higher the educational level, the higher the chances of health awareness, since parents with university- and postgraduate-level education showed significantly greater awareness than illiterate participants [14]. Moreover, a recent Saudi study by Almalki et al. involving 1023 parents of children aged 14 years or younger found that parents' knowledge of eye health in children was significantly correlated with high educational attainment (*p* < 0.001), since holders of PhD degrees had better knowledge of eye care than those with Master's degrees (*p* < 0.001), diplomas (*p* < 0.001), and Bachelor's degrees (*p* < 0.001) [18]. This finding has also been reported in other studies [[15], [16], [17],^19^], and it may be related to the fact that well-educated parents are more likely to seek healthcare facilities and obtain information regarding childhood eye diseases and eye care from qualified personnel. In contrast, a descriptive study conducted in Swaziland, Southern Africa, found no significant association between parents' level of education and their knowledge of childhood eye conditions (*p* = 0.112) [20]. This outcome was consistent with the findings obtained by Baashar et al. who showed that Saudi parents' awareness and knowledge of children's eye care was below average regardless of their level of education [5]. Nevertheless, the sample size in the previous study was too small to reach any statistical significance. The current study also found that working in a healthcare institution was associated with a higher knowledge level of pediatric eye diseases and care among parents (*p* < 0.0001). This finding is quite logical. Parents who work in the medical field can be expected to have an above-average level of knowledge about childhood eye conditions and may stay up to date with the latest eye care recommendations for children. Moreover, our findings showed that having a child with an eye disease (*p* = 0.001) was an important factor contributing to parents' increased knowledge of common pediatric eye conditions and their management. This is consistent with the results of another study conducted in Madinah, Saudi Arabia [12]. This observation was also reported in other studies [15,17]. The higher level of knowledge in these parents is probably related to their frequent contact with professional eye care practitioners as part of their children's management. In addition, parents of children with established eye diseases are often concerned about having another child with an eye disease and therefore focus more on understanding the causes of eye diseases and measures for prevention.

### 4.2 Parents' attitudes regarding children's eye diseases

Most of the participating parents in this study found the use of corrective spectacles for their children's eye health highly acceptable (92.4 %). Similarly, in a previous study conducted in the Aseer region of Saudi Arabia, only 23.6 % of surveyed parents reported that they would be upset if their children wore eyeglasses [16]. On the other hand, Bashaar et al. reported an acceptance rate of only 42.3 % among the tested parents [5]. Notably, the present study showed a higher parental acceptance rate for eyeglass usage in children than those reported in other local studies, including those in Arar (73 %), Madinah (76.9 %), and Makkah (81.1 %) [12,15,17]. This improved attitude toward optical correction reflects the increased awareness of the rising prevalence of refractive errors and their impact on children among Saudi parents. Our data also showed that parents with an educational level of high school or lower were more likely to reject corrective spectacle use (*p* < 0.0001). This finding is also consistent with the results reported in the literature [14,20].

Among the few parents (7.6 %) who refused the use of corrective glasses, fear of limiting the child's daily activities and concerns about their cosmetic appearance were the most frequently reported reasons. Similarly, the study conducted in Arar found that most mothers did not accept spectacles for their children because spectacles may limit their children's daily activities as well as for cosmetic reasons or social stigma [17]. Another study conducted in Nigeria to assess the factors influencing the eye care-seeking behavior of parents for their children stated that most parents were hesitant to get glasses for their children because they feared that the glasses may be harmful when worn over a long time [22]. However, Alrasheed et al. aimed to assess Saudi parents' awareness of and perspectives on childhood refractive errors and found that 70.1 % believed that wearing eyeglasses would not deteriorate their children's vision over time, which was statistically significant (*p* = 0.005) [21].

In this study, only 48 % of parents utilized government healthcare services for their children's eye diseases, and 69 % of the parents who utilized these services expressed satisfaction with them. This aligns with the satisfaction rate of 67.7 % reported in a previous study in Arar City [17], indicating consistency across various regions. Most parents involved in this study (83.2 %) expressed their willingness to attend educational sessions on childhood eye diseases and care whenever such sessions were available. In contrast, the Arar study reported that only 26.6 % of the participants were willing to attend future educational sessions on children's eye diseases [17]. This difference may be attributed to differences in demographic data among the studied populations. The parents' willingness to attend these sessions reflects their cooperation in improving eye care for their children and their desire to obtain health information from trusted sources. This finding indicates the need for the Saudi government to put extra effort into arranging educational sessions and awareness campaigns, and making them available to all parents. These measures will be beneficial in reducing the burden of childhood visual impairment and blindness in the country over the long term.

### 4.3 Parents' practices regarding children's eye diseases

The most frequently reported reasons for which parents considered taking their children for eye examinations were “advice from a health care provider,” and “concerns about poor vision.” This supports the results of a previous study conducted in Saudi Arabia, which reported that concerns about poor vision were the most common reason for which parents sought eye care for their children [18]. Consistent with this finding, in a study involving young children in London, more than two-thirds (67 %) of the surveyed parents selected “concerns about poor vision” as a reason that prompted them to seek an eye test for their child [13].

Approximately two-thirds (65.6 %) of the parents participating in this study did not take their children for periodic eye examinations. Furthermore, over one-third of the tested parents (37.5 %) had not taken their children for eye examinations at 6 months of age. In a study conducted in Ghana, Honduras, and India, Ramai et al. found that while 95 % of parents believed in the importance of eye examinations for their children, 66 % responded that none of their children had ever received an eye exam [23]. In Saudi Arabia, a study on amblyopia awareness among parents also showed that the majority (60 %) of participants had not taken their children for a comprehensive eye examination [24]. Consistent with these findings, Almalki et al. noted that the majority (60.9 %) of parents had not taken their children for any form of eye or vision testing [18]. Similarly, a study conducted in the Aseer region of Saudi Arabia reported that more than half of the respondents (51.5 %) had not taken their children for an eye examination [16]. In the published literature, the perception that an eye examination was not needed was one of parents’ top reasons for not seeking eye care for their children. For example, in a Nigerian study, most parents (60 % of fathers and 57 % of mothers) believed that routine eye examinations were unnecessary for their children [25]. Nirmalayan et al. found that respondents did not see the need for periodic eye examinations unless the child complained or the parents identified a problem [26]. In a local study, Bashaar et al. reported that 61.9 % of parents found eye examinations unnecessary [5]. These findings are consistent with those of previous studies [12,16].

Another Nigerian study found that parents were more likely to take their children for an eye test when they showed noticeable symptoms and had repeated complaints than for conditions that they could not see or perceive [22]. Ramai et al. also noted that most parents in Ghana, Honduras, and India perceived eye care as important only upon the manifestation of visible or perceived visual impairment [23]. Similarly, a study performed in Selangor, Malaysia, reported that parents would seek eye care professionals' help only when their children had signs of vision problems, such as rubbing their eyes constantly, sitting very close to the television, or holding the book too close to their face when they read [19]. These findings are consistent with the outcomes of the present study, which demonstrated that most parents (40 %) do not seek an eye examinations for their children because they do not have an eye problem. Parents often question the need for a regular eye check-up if their children's eyes look “normal” and they do not have any complaints regarding vision. This can be attributed to a lack of parental knowledge regarding the importance of periodic eye examinations in detecting vision problems at an early age, so that effective management can take place before it is too late. However, in this study, working in the healthcare industry and having a child with an eye disease was shown to raise awareness of the importance of such checkups among parents (*p* = 0.013 and < 0.0001, respectively). This is mainly because these parents have a better understanding of the impact of poor vision on their children, since they visit the hospital more often and are likely to obtain information from professional personnel. Interestingly, this study found no significant association between parents' educational levels and their tendency to take their children for periodic eye examinations. This result differs from that of a study by Kimel, which showed that children who received eye examinations had parents with at least a diploma [27]. Moreover, Almalki et al. noted that parents with an educational level less than high school were significantly more likely to face barriers to seeking eye care [17]. These findings could be attributed to the fact that most people with higher education were higher earners and could afford healthcare services.

Other barriers that prevent parents from taking their children for periodic eye examinations include not knowing how and/or where to access appropriate eye care (16 %), lack of time (15 %), believing that their children were too young to have their eyes tested (12 %), and worries about testing and treatment expenses (10 %), all of which have been identified, with some differences in order, by previously published studies [13,16,18,22,25].

In the present study, when parents had concerns about their children's eye care, 30 % only trusted doctors in clinics as their source of information. However, a total of 40 % of the participants relied on the Internet and social media to answer their questions regarding children's eye care. In a similar study, family doctors and social media were the main sources of information on child eye care for 37.1 % and 22.7 % of the participants, respectively [5]. While these results have been confirmed in another study [12], some studies have reported that the primary sources of information are community members such as friends and family [14,28,29]. This finding was attributed to the idea that people tend to accept shared experiences or advice, albeit inaccurate or non-evidence-based, from someone they know regardless of whether they were healthcare professionals or not [13]. Nevertheless, the results highlight the need for increased parental education regarding reliable sources of information on pediatric eye needs.

This study had some limitations. Its cross-sectional design only proved associations and did not clarify causality, and its online approach and convenience sampling may have affected the generalizability of the results. In addition, the study only tested the level of parental knowledge about children's eye care in general. Further studies are required to test the knowledge level of Saudi parents about specific eye conditions in children, such as strabismus, glaucoma, and cataracts. Saudi Arabia is a large country with many regions and cities, and an extensive study with a large sample size is needed for accurate results. Nevertheless, an important strength of this study was that it was conducted with a reasonably large number of participants from each region.

## 5 Conclusions

This study indicated that the level of parental knowledge of children's eye care in Saudi Arabia was acceptable. The belief that routine eye checkups are unnecessary in the absence of eye complaints reflects a poor parental understanding of the impact of poor vision on children. Additionally, all parents should be informed of how, when, and where they can access eye care for their children, and of existing screening programs. Overall, these data indicate the need for proper parental education by professional personnel to optimize children's eye healthcare.

## CRediT authorship contribution statement

**Sokinah N. Al Musalami:** Writing – original draft, Supervision, Formal analysis, Data curation, Conceptualization. **Reem J. Al Qasim:** Writing – original draft. **Bayan S. Alshuhayb:** Writing – review & editing, Resources, Methodology, Conceptualization. **Abdulaziz I. Al-Somali:** Supervision.

## Ethical consideration

All participants gave their consent. All data were kept confidential and used only for research purposes.

## Data availability statement

The study data is available from the corresponding author for reasonable request.

## Funding

There was no funding for this study.

## Declaration of competing interest

The authors declare that they have no known competing financial interests or personal relationships that could have appeared to influence the work reported in this paper.

## References

[1] Gilmore J.H., Knickmeyer R.C., Gao W. (2018). Imaging structural and functional brain development in early childhood. Nat. Rev. Neurosci..

[2] World Health Organization (December 8, 2017). Vision impairment and blindness. https://www.who.int/news-room/fact-sheets/detail/blindness-and-visual-impairment.

[3] Parrey M.U.R. (2019). Prevalence and causes of visual impairment in Saudi children of Arar city. The Annals of Clinical and Analytical Medicine.

[4] Strategic Plan for Vision 2020: The Right to Sight. n.d. https://apps.who.int/iris/bitstream/handle/10665/205887/B5084.pdf?sequence=1. Accessed October 13, 2023.

[5] Baashar A., Yaseen A., Halawani M., Alharbi W., Alhazmi G., Alam S., Eldin E. (2020). Parents knowledge and practices about child eye health care in Saudi Arabia. International Journal of Medicine in Developing Countries.

[6] Al-Rowaily M.A. (2010). Prevalence of refractive errors among pre-school children at king abdulaziz medical city, Riyadh, Saudi Arabia. Saudi J Ophthalmol.

[7] Aldebasi Y.H. (2015). Prevalence of amblyopia in primary school children in Qassim province, Kingdom of Saudi Arabia. Middle East Afr. J. Ophthalmol..

[8] Bardisi W.M., Bin B.S. (2002). Vision screening of preschool children in Jeddah, Saudi Arabia. Saudi Med. J..

[9] Al-Tamimi E.R., Shakeel A., Yassin S.A., Ali S.I., Khan U.A. (2015). A clinic-based study of refractive errors, strabismus, and amblyopia in pediatric age-group. J Family Community Med.

[10] Challa N.K. (2022). Prevalence of amblyopia among the children of Saudi Arabia: a systematic review, 1990–2020. African Vision and Eye Health.

[11] Alghamdi W. (2022). Prevalence of refractive errors among children in Saudi Arabia: a systemic review. Open Ophthalmol. J..

[12] Surrati A.M. (2022). Parents' awareness and perception of children's eye diseases in Madinah, Saudi Arabia: a cross-sectional study. Cureus.

[13] Donaldson L. (2018). Eye care in young children: a parent survey exploring access and barriers. Clin. Exp. Optom..

[14] Al Mazrou A. (2021). Do Saudi parents have sufficient awareness of pediatric eye diseases in Riyadh?. Saudi journal of ophthalmology.

[15] Almogbel A.H. (May 01, 2023). Parents' awareness and attitude toward pediatrics eye diseases in Makkah, Saudi Arabia: a cross-sectional study. Cureus.

[16] Aldhabaan W. (2022). Knowledge and practices of child eye healthcare among parents in aseer region, Saudi Arabia. Cureus.

[17] Parrey M.U.R. (2019). Parents' awareness and perception of children's eye diseases in Arar City. The Annals of Clinical and Analytical Medicine.

[18] Almalki A. (2022). Parent's eyecare seeking behavior for young children in Saudi Arabia. Saudi J. Health Sci..

[19] Wan Omar WE., Abdul Razak N.A. (2020). https://healthscopefsk.com/index.php/research/article/view/95.

[20] Sukati V.N. (2018). Knowledge and practices of parents about child eye health care in the public sector in Swaziland. African journal of primary health care & family medicine.

[21] Alrasheed S.H., Alghamdi W.M. (2022). Parents' awareness of and perspectives on childhood refractive error and spectacle wear in Saudi Arabia. Sultan Qaboos University medical journal.

[22] Ebeigbe J.A. (2018). Factors influencing eye-care seeking behaviour of parents for their children in Nigeria. Clin. Exp. Optom..

[23] Ramai D., Elliott R., Goldin S., Pulisetty T. (2015). A cross-sectional study of pediatric eye care perceptions in Ghana, Honduras, and India. Journal of Epidemiology and Global Health.

[24] Alsaqr A.M., Masmali A.M. (2019). The awareness of amblyopia among parents in Saudi Arabia. Therapeutic advances in ophthalmology.

[25] Amiebenomo O., Achugwo D., Abah I. (2016). Parental knowledge and attitude to children's eye care services. Niger. J. Paediatr..

[26] Nirmalayan P., Sheeladevi D., Tamilselvi S., Victor A., Vijayalakshmi P., Rahmathulla L. (2004). Perception of eye disease and eye care needs of children among parents in rural South India: the Kariapatti pediatric eye evaluation project (KEEP). Comm Eye Care.

[27] Kimel L. (2006). Lack of follow-up exams after failed school vision screenings: an investigation of contributing factors. J. Sch. Nurs..

[28] Al Zarea B.K. (2016). Assessment of awareness, knowledge, attitudes and practices associated with eye diseases in the population of Aljouf and Hail Province of Saudi Arabia. Int. J. Med. Res. Prof..

[29] Sultan I., Alsaedi M., Ahmed F. (2019). Knowledge and awareness of age related eye diseases in the population of the Western region of Saudi Arabia. World Fam Med J.

